# Increased cross-presentation by dendritic cells and enhanced anti-tumour therapy using the Arp2/3 inhibitor CK666

**DOI:** 10.1038/s41416-022-02135-4

**Published:** 2023-01-11

**Authors:** Mariana M. S. Oliveira, Roberta D’Aulerio, Tracer Yong, Minghui He, Marisa A. P. Baptista, Susanne Nylén, Lisa S. Westerberg

**Affiliations:** 1 Department of Microbiology Tumor and Cell biology, Karolinska Institutet, 17177 Stockholm, Sweden; 2 Institute for Virology and Immunobiology, Julius-Maximilians-University Würzburg, Würzburg, Germany

**Keywords:** Immunotherapy, Oncology

## Abstract

**Background:**

Dendritic cell (DC) vaccines for cancer therapy offer the possibility to let the patient’s own immune system kill cancer cells. However, DC vaccines have shown less efficacy than expected due to failure to induce cancer cell killing and by activating T regulatory cells.

**Methods:**

We tested if inhibition of signalling via WASp and Arp2/3 using the small molecule CK666 would enhance DC-mediated killing of tumour cells in vitro and in vivo.

**Results:**

Using CK666 during the ex vivo phase of antigen processing of ovalbumin (OVA), murine and human DCs showed decreased phagosomal acidification, indicating activation of the cross-presentation pathway. When compared to untreated DCs, DCs treated with CK666 during uptake and processing of OVA-induced increased proliferation of OVA-specific CD8^+^ OT-I T cells in vitro and in vivo. Using the aggressive B16-mOVA melanoma tumour model, we show that mice injected with CK666-treated DCs and OVA-specific CD8^+^ OT-I T cells showed higher rejection of B16 melanoma cells when compared to mice receiving non-treated DCs. This resulted in the prolonged survival of tumour-bearing mice receiving CK666-treated DCs. Moreover, combining CK666-treated DCs with the checkpoint inhibitor anti-PD1 further prolonged survival.

**Conclusion:**

Our data suggest that the small molecule inhibitor CK666 is a good candidate to enhance DC cross-presentation for cancer therapy.

## Introduction

Dendritic cells (DCs) can acquire exogenous antigens derived from a pathogen and present them in major histocompatibility complex (MHC) class I molecules in a process called cross-presentation [1]. Although various types of cells can cross-present model antigens in vitro, studies indicate that DCs are the main cross-presenting cell in vivo [2, 3]. Two main pathways regulate cross-presentation by DCs, the cytosolic and vacuolar pathways [4–6]. The cytosolic pathway is sensitive to proteasome inhibitors and is dependent on transporters associated with antigen processing (TAP). The exogenous antigen is stored in early endosomes or phagosomes, transported to the cytoplasm by endoplasmic reticulum (ER) associated degradation (ERAD) members and degraded by the proteasome in the cytosol. After degradation, the peptides are transported by TAP to the ER to be loaded on MHC class I molecules [4, 7, 8]. The vacuolar pathway is independent of TAP and proteasome and is insensitive to protease inhibitors, in particular to cathepsin S inhibitors [9, 10]. The exogenous antigen is degraded by lysosomal proteases and loaded into endocytic compartments [11–13]. An unmet need in anti-cancer immunotherapy is to enhance the cross-presentation of tumour antigens by DCs for the activation of cytotoxic CD8^+^ T cells [14, 15].

We recently showed that murine DCs lacking Wiskott-Aldrich syndrome protein (WASp) favour the cross-presenting pathway for exogenous antigen. WASp-deficient spleen DCs, both CD8^+^ and CD8^−^ DCs, and bone marrow (BM)-derived DCs have decreased phagosomal acidification and maintain a neutral pH of the phagosome. This led to enhanced cross-presentation and exogenous antigen is presented on MHC class I molecules, rather than on MHC class II molecules, resulting in increased proliferation of CD8^+^ T cells [16]. WASp is a hematopoietic-expressing protein that is responsible for maintaining DCs cytoarchitecture [17]. DCs that lack WASp are unable to polarise normally and show severely reduced translocational motility in vitro [17]. WASp was found to be essential for DC podosome formation [18]. DCs lacking WASp have defects in attachment and detachment on fibronectin-coated slides, reduced migratory capacity of Langerhans cells from the skin to the draining lymph nodes using fluorescein isothiocyanate (FITC) and oxazolone contact hypersensitivity models [18, 19]. The domain structure of WASp comprises a carboxyl terminus, consisting of the verpolin homology (V) domain, the cofilin homology (C) domain and the acidic region (A) termed the VCA domain. The VCA domain is largely conserved among WASp family members and binds a complex formed of actin-related proteins (Arp), the Arp2/3 complex, that recruits monomeric actin to polymerise new actin filaments [20, 21]. At steady state, WASp is in an inactive autoinhibited conformation that opens upon the interaction of the small Rho GTPase Cdc42 with the WASp GTPase binding domain (GBD), leaving the VCA domain exposed for actin filaments assembly [22–24].

DC uptake, antigen processing and presentation can be modulated in order to improve DC-mediated tumour therapy. Different actin regulators coordinate specific cell responses and it is possible that inhibition of specific actin regulators may be beneficial in supporting different stages of DC maturation and their functionality [25]. CK666 is an Arp2/3 inhibitor that stabilises the inactive state of the complex, blocking the movement of the Arp2 and Arp3 subunits into the activated filament-like (short pitch) conformation [26]. Wiskostatin is an inhibitor of the WASp homologue N-WASp that binds within a pocket in the GBD regulatory domain that maintains N-WASp and WASp in an inactive, autoinhibited conformation [27]. ML141 is a Cdc42 inhibitor that is reported as a selective and non-competitive allosteric inhibitor, although the mechanism of action is not completely understood yet [28, 29]. These inhibitors are used to impair actin-based processes and have been tested for their action in killing cancer cells. CK666 inhibits the migration and invasion of glioma cells [30] and inhibits the migration and spreading of HeLa cervical cancer cell line and U2OS osteosarcoma cell line [31]. Wiskostatin significantly inhibits cell growth as well as motility, migration and adhesion of A-549 and SK-MES-1 lung cancer cell lines [32] and reduces the migration of MDA-MB-231 breast cancer cells [33]. ML141 inhibits the migration of OVCA429 ovarian cancer cells [29]. For immunotherapy, as tested in this study, an advantage of these inhibitors is that their activity is reversible and they can be washed away [34].

One promising immunotherapy, applied for aggressive melanoma, is the use of autologous tumour-specific T cells. T cells are isolated from the patient peripheral blood, expanded ex vivo together with patient PBMCs and digested tumour, and thereafter reinfused back into the patient. This technique results in persistent clonal repopulation of T cells with no serious toxicity observed and, importantly, regression of the tumour in the majority of the patients [35–38]. In these approaches, T-cell priming is mainly controlled by DCs. Therefore, there have been quite some efforts to establish DC-based cancer therapy; however, some DC-based approaches have low or no efficacy and the overall response rate is ~3% [39]. The low success rate is believed to be, in part, because DCs do not only activate CD8^+^ cytotoxic T cells but also activate CD4^+^ T regulatory cells that are unfavourable for tumour killing [40]. Despite these challenges, DC-based induction of “poised” T cells is a promising approach for tumour therapy [41].

In this study, we tested if we could promote DC cross-presentation for DC-based immunotherapy by treating DCs with small molecule inhibitors for WASp signalling proteins. Using the Arp2/3 inhibitor CK666, DCs showed minimal toxicity during 16 h of exposure. Using acidification and proliferation assays, we show that both mouse and human CK666-treated DCs decreased phagosome acidification and that murine DCs induced increased proliferation of cytotoxic CD8^+^ T cells in vitro and in vivo. Mice bearing the aggressive B16-mOVA melanoma tumour and receiving CK666-treated DCs and OVA-specific CD8^+^ T cells showed prolonged survival when compared to mice receiving untreated DCs. Our data suggest that small molecule inhibitor CK666 is a good candidate to modulate DC cross-presentation to enhance the killing of tumour cells.

## Materials and methods

### Human samples

Peripheral blood samples were obtained from purchased anonymized by-products of blood donations from healthy adult donors at the Karolinska University Hospital Blood Bank.

### Mice

All animals used; CD45.2 WT, OT-I and CD45.1 (Ly5.1) mice on C57Bl/6 background were bred and maintained at the animal facility of the Comparative Medicine Wallenberg (KMW) and Comparative Medicine Annex (KMA) at Karolinska Institutet under specific pathogen-free conditions. Mice were used at 8–13 weeks of age. All animal experiments were performed according to the EU Directive 2010/63/EU for animal experiments and after approval from the local ethical committee (the north Stockholm district court, permits #N272/14 and #11159-2018, PI: L.S.W) and in accordance with national and institutional guidelines.

### Cell culture

Murine bone marrow-derived DCs (BM-DCs) from C57Bl/6 mice were obtained by culturing bone marrow precursors for 6–7 days in RPMI medium (Sigma–Aldrich) containing 10% FBS decomplemented and filtered (Gibco), 20 mM L-glutamine (Gibco), 100 U/ml penicillin–streptomycin (Gibco), 50 µM 2-mercaptoethanol (Gibco) and 20 ng/ml of murine granulocyte-macrophage colony-stimulating factor (GM-CSF) in non-tissue culture petri dishes (Sarstedt). A new medium with murine GM-CSF was added on day 3 of culture. Immature DCs were obtained by gentle recovery of semi-adherent cells from culture dishes. Human monocyte-derived DCs (moDCs) were obtained by isolating PBMCs from buffy coat samples obtained from anonymous blood donors at the Karolinska University Hospital. Total PBMCs were let to adhere in T150 tissue culture flasks (Sarstedt) for 2 h at 37 °C. The supernatant containing lymphocytes was removed and monocytes, adhered to the flasks, where washed once with PBS and cultured for 6–7 days in a complete RPMI medium containing 10% FBS decomplemented and filtered (Gibco), 20 mM L-glutamine (Gibco), 100 U/ml penicillin–streptomycin (Gibco), 50 µM 2-mercaptoethanol (Gibco) and 60 ng/ml of human GM-CSF for 3 days. On day 3, more medium with human GM-CSF and 50 ng/ml human IL-4 was added to the culture. Immature DCs were obtained by gentle recovery of semi-adherent cells from culture flasks.

### Survival curve, actin content and TLR4 expression

Immature mouse BM-DCs or immature human moDCs were culture in a 96-well tissue culture-treated plate (Sarstedt) for 16 h with concentrations of CK666 from 0 to 40 µM. Cell viability was assessed by FACS after staining the cells with Live/Dead Fixable Aqua Dead Cell Stain (Invitrogen). Actin content and TLR4 expression were examined by staining DCs with phalloidin-Alexa488 (Invitrogen) and TLR4 (Biolegend; SA15-21) on fixed and permeabilized cells (BD cytofix/cytoperm kit).

### Acidification assay

To assess acidification capacity, ovalbumin (Sigma–Aldrich) was linked to pH-rodo (Invitrogen) according to the manufacturer’s instructions. To control the amount of ovalbumin-pHrodo taken up over time by each cell, a particulate antigen assay was performed by coating 3 µm latex beads (Life Technologies) with pHrodo-ovalbumin. DCs were incubated with 50 µg /ml ovalbumin-pH-rodo for 30 min (mouse BM-DCs) or 16 h (human moDCs). DCs that took up one bead were gated on using flow cytometer parameters FSC vs SSC for acidification analysis. Upon incubation, cells were stained with CD11c (eBioscience; clone #N418), MHC class II (Biolegend; clone #M5/114.15.2) and CD8 (BD Biosciences; clone #53-6.7)—mouse BM-DCs; or CD1c (Biolegend; clone #L161) and CD14 (Biolegend; clone #HCD14)—human moDCs.

### In vitro CD8+ T-cell proliferation

BM-DCs from wildtype C57Bl/6 mice were differentiated and on day 6, DCs were treated with 10 µM CK666 (Sigma–Aldrich) for 1 h before different concentrations of OVA (Sigma–Aldrich) or 2 µg/ml of SIINFEKL (GenScript) and 500 ng/ml LPS (*Salmonella enterica* serotype *typhimurium*; Sigma–Aldrich) were added to the culture. BM-DCs were incubated for 5 more hours, resulting in exposure to CK666 for a total of 6 h. CD8^+^ T cells were isolated from the spleen of OT-I transgenic mice by negative selection using the CD8α T-cell isolation kit (Miltenyi Biotec). The CD8^+^ T-cell population of purity above 95%, was labelled with CFSE (Invitrogen) by incubating 10^7^ cells with 2 µM CFSE for 10 min at 37 °C. The reaction was stopped by adding a complete RPMI medium to the cells. Both T cells and BM-DCs were washed 3–4 times in Dulbecco’s Phosphate Buffered Saline (DPBS) (HyClone) to remove all CFSE and CK666 inhibitor; and re-counted. BM-DCs were co-cultured with CFSE-labelled CD8^+^ OT-I T cells at 1:10 DC:T-cell ratio in a U-bottom tissue culture 96-well plate for 48 h and analysed by FACS. Antibodies used: Live/Dead Fixable Aqua Dead Cell Stain (Invitrogen), CD3 (Biolegend; clone #17A2), CD8 (BD Biosciences; clone #53-6.7), CD69 (Biolegend; clone #H1.2F3), IFNγ (Biolegend; clone #XMG1.2), IL-2 (Biolegend; clone #JES6-5H4), CFSE (Invitrogen). In experiments where cells were stimulated, 50 ng/ml of phorbol 12-myristate 13-acetate (PMA; Sigma–Aldrich), 1 µg/ml of ionomycin (Sigma–Aldrich) and 1 µl/ml of GolgiPlug protein transport inhibitor (BD Biosciences) were added to the last 4 h of the culture.

### In vivo migration assay

BM-DCs from CD45.1 (Ly5.1) were cultured, left without treatment or treated with CK666, plus OVA and LPS as above. Cells were washed 3–4 times in DPBS (HyClone) before injection into mice. Twenty microlitres of mature BM-DCs was injected in each footpad of CD45.2 WT mice in a total of 1 × 10^6^ cell/footpad. After 48 h, popliteal dLNs were collected, incubated with collagenase type 3 (Worthington) for 45 min at 37 °C before being mashed in single-cell suspension and stained for FACS for analysis. Antibodies used: Live/Dead Fixable Aqua Dead Cell Stain (Invitrogen), CD8 (BD Biosciences; clone #53-6.7), CD11c (eBioscience; clone #N418), MHCII (Biolegend; clone #M5/114.15.2), CD45.1 (Biolegend; clone #A20), CD45.2 (Biolegend; clone #104).

### In vivo CD8+ T-cell proliferation

BM-DCs from CD45.1 (Ly5.1) were cultured and CD8^+^ OT-I T cells isolated and labelled with CFSE as above. Cells were washed 3–4 times in DPBS (HyClone) before being injected into CD45.2 WT mice. Twenty microlitres of mature BM-DCs was injected in each footpad of CD45.2 WT mice in a total of 1 × 10^6^ cell/footpad. 2.5 × 10^6^ CD8^+^ OT-I T cells/mouse were injected intravenously. After 48 h, popliteal dLNs were collected, incubated with collagenase type 3 (Worthington) for 45 min at 37 °C before being mashed in single-cell suspension and stained for FACS for analysis. Antibodies used: Live/Dead Fixable Aqua Dead Cell Stain (Invitrogen), CD3 (Biolegend; clone #17A2), CD4 (Invitrogen; clone #RM4-5), CD8 (BD Biosciences; clone #53-6.7), CD11c (eBioscience; clone #N418), CFSE (Invitrogen)

### B16-mOVA rejection

B16-mOVA cell line was selected with geneticin G418 (Gibco) for 2 weeks before use. All tumour experiments were performed with the same passage number of the cell line. 150,000 B16-mOVA cells were injected subcutaneously 1:1 in Matrigel® Basement Membrane Matrix, phenol red-free (Corning). After 14 days, CK666-treated or non-treated BM-DCs previously primed with OVA (Sigma–Aldrich) and activated with LPS (Sigma–Aldrich) were injected in the footpad; and CD8^+^ OT-I T cells were injected intravenously in the tail vein. The tumour volume was measured using a digital caliper every 2 days, and mice were sacrificed when the tumour reached the volume of 1 cm^3^. Tumour volume was calculated by assuming that the tumour has an ellipsoid shape (cm^3^): (l × w^2^)/2, where l (length) is the larger of two perpendicular axes and w (width) is the smaller of two perpendicular axis. When α-PD1 (BioXCell; cat #BE0146) was used, 150 µg of the protein in a DPBS solution was injected together with CD8^+^ T cells intravenously.

## Results

### Treatment of DCs with small molecule inhibitor CK666 leads to decreased phagosomal acidification and increased CD8^+^ T-cell proliferation

**Fig. 1:**
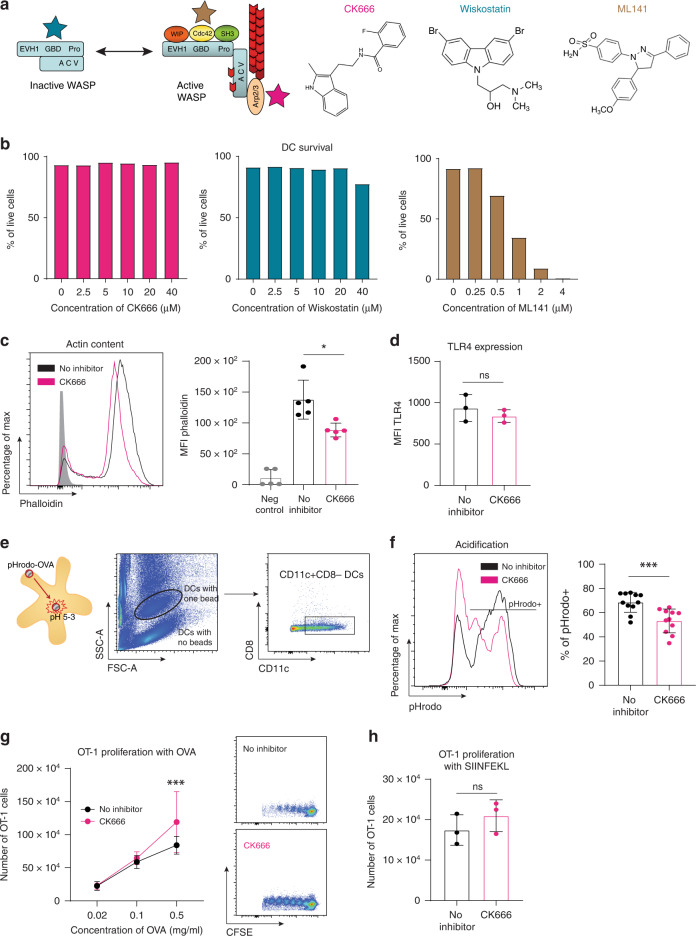
Treatment of BM-DCs with small molecule inhibitor CK666 leads to decreased phagosomal acidification and increased CD8^+^ T-cell proliferation. **a** WASp in inactive and active conformation and the location where each inhibitor acts. CK666 (pink star) inhibits the Arp2/3 complex; Wiskostatin (blue star) inhibits WASp open conformation; and ML141 (brown star) inhibits Cdc42. Structural formula for CK666 (pink), wiskostatin (blue) and ML141 (brown) inhibitors. **b** Survival curve for 16 hours of BM-DCs treated with CK666 (pink) and wiskostatin (blue) with concentrations between 0 and 40 µM and ML141 (brown) with concentrations between 0 and 4 µM. **c** Actin content of CK666-treated BM-DCs (pink line) or non-treated BM-DCs (black line) compared to a negative control (grey filled line) and quantification. Results represent pooled data from 3 different experiments with 5 different animals. Each dot represents the average of 3 technical replicates. **d** TLR4 expression on non-treated BM-DCs (black) and CK666-treated BM-DCs (pink). Results represent pooled data from 1 experiment with 3 different animals. Each dot represents the average of 3 technical replicates. **e** Gating strategy used to assess acidification on CK666-treated and non-treated BM-DCs. **f** Representative acidification plot by CK666-treated (pink line) or non-treated BM-DCs (black line) and quantification of pHrodo^+^ cells. Results represent pooled data from 3 different experiments using 3 different animals and 3–4 technical replicates per animal. **g**, **h** OT-1 cell proliferation induced by CK666-treated (pink line) or non-treated (black line) BM-DCs that were given in **g**; different amounts of OVA from 0.02 to 0.5 mg/ml or in **h**; SIINFEKL 2 µg/ml. Each dot corresponding to OVA concentration represents data pooled from 3 to 5 different experiments with 3 technical replicates each. Graphs show mean values±SD and significance was assessed by unpaired t-test; ordinary one-way ANOVA with multiple comparisons; or two-way ANOVA with multiple comparisons. *p ≤ 0.05, **p ≤ 0.01, ***p ≤ 0.001, ****p ≤ 0.0001. Representative flow cytometry plots of the OT-1 cell proliferation peaks with non-treated BM-DCs or CK666-treated DCs, based on CFSE staining, are shown.

We have previously showed that both splenic and BM-DCs that lack WASp have decreased phagosomal acidification, which results in increased cross-presentation and proliferation of cytotoxic CD8^+^ T cells [16]. We here tested if we could mimic increased cross-presentation by WASp-deficient DC using small molecule inhibitors of WASp signalling pathways (Fig. 1a). We tested the small molecule inhibitors for Arp2/3 (CK666) [26], (N-)WASp (Wiskostatin) [27] and Cdc42 (ML141) [28] and we reasoned that DCs should be exposed to these inhibitors during the antigen uptake and processing phase to enhance cross-presentation and then washed away. We first tested DC viability upon exposure to a dose range of inhibitors. BM-DCs were treated for up to 16 h with 0–40 µM for CK666 and Wiskostatin and 0–4 µM for ML141 (Fig. 1b). The ML141 inhibitor revealed to be very toxic to BM-DCs whereas both CK666 and Wiskostatin were well tolerated by BM-DCs. For all time points, BM-DC viability was greater than 90%. CK666 inhibits Arp2/3 mediated actin filaments assembly, and for this reason, we next examined F-actin content by flow cytometry. DCs treated with CK666 had lower F-actin content when compared to non-treated BM-DCs (Fig. 1c). To examine if reduced F-actin of CK666-treated DCs would influence cell surface expression of DC maturation molecules, we measured the receptor TLR4. Cell surface expression of TLR4 were similar between non-treated BM-DCs and CK666-treated BM-DCs (Fig. 1d). We chose to continue working with the CK666 inhibitor since it has been well characterised by interaction within the pocket between the Arp2 and Arp3 subunits of the Arp2/3 crystal structure [26, 42], and decreased F-actin polymerisation at 10 µM without affecting DC viability or TLR4 cell surface expression (Fig. 1b–d). We next performed functional assays for CK666-treated and non-treated BM-DCs. Decreased phagosomal acidification leads to the escape of the antigen to the cytoplasm causing it to be presented at MHC class I molecules instead of MHC class II molecules. To measure acidification, BM-DCs were treated with CK666 for 1 h before 3 µm latex beads coated with the pH sensor pHrodo were added to the culture (Fig. 1e). This allows the activity of the inhibitor before bead uptake by the BM-DCs. BM-DCs were co-cultured with pHrodo-coated beads for 30 more minutes in the presence of CK666, and then analysed by flow cytometry. To compare the phagosomal capacity, the population was pre-gated on BM-DCs that had phagocytosed one bead as shown in the gating strategy (Fig. 1e). BM-DCs treated with CK666 showed decreased acidification compared to non-treated BM-DCs (Fig. 1f). To test the capacity to cross-present ovalbumin, CK666-treated and non-treated DCs pulsed were co-cultured with CD8^+^ OT-1 T cells for 48 h. CK666-treated DCs induced increased proliferation of CD8^+^ OT-1 T cells when compared to non-treated DCs using 0.5 mg/ml OVA (Fig. 1g). Non-treated BM-DCs and CK666-treated BM-DCs loaded with the SIINFEKL-OVA peptide induced similar CD8^+^ OT-1 T-cell proliferation (Fig. 1h), suggesting a specific effect of CK666 treatment on antigen processing rather than peptide loading of DCs. Together, these results show that CK666-treated BM-DCs have decreased phagosomal acidification, resulting in increased CD8^+^ T-cell proliferation.

### Human monocyte-derived DCs treated with CK666 show decreased phagosomal acidification

**Fig. 2:**
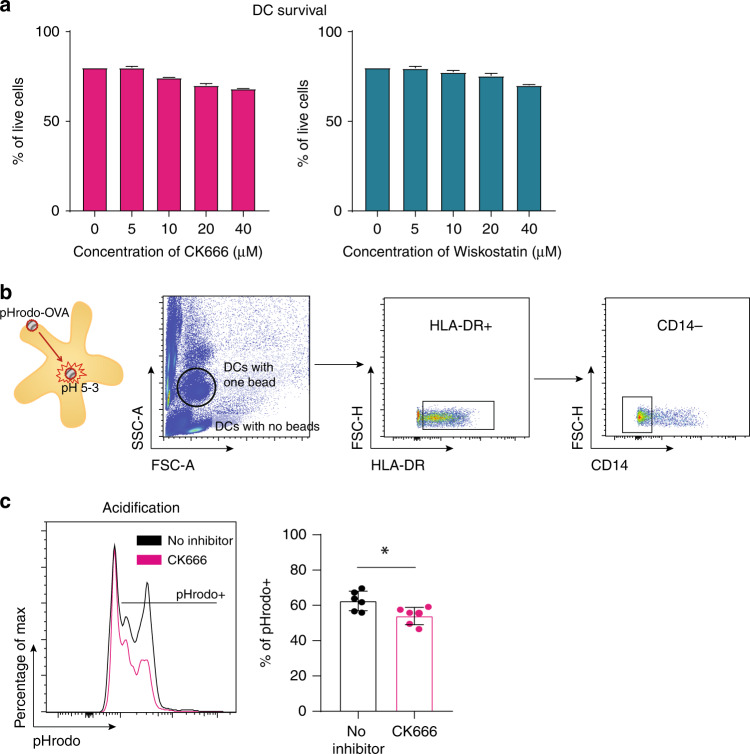
Human monocyte-derived DCs treated with CK666 show decreased phagosomal acidification. **a** Survival curve for 16 h of moDCs treated with CK666 (pink) and Wiskostatin (blue) with concentrations between 0 and 40 µM. **b** Gating strategy used to assess acidification on CK666-treated and non-treated moDCs. **c** Representative acidification plot by CK666-treated (pink line) or non-treated moDCs (black line) and quantification of pHrodo^+^ cells. Results represent pooled data from 3 different experiments using 3 different donors and 2 technical replicates per donor. Graphs show mean values ± SD and significance was assessed by unpaired *t*-test. *p ≤ 0.05, **p ≤ 0.01, ***p ≤ 0.001, ****p ≤ 0.0001.

We next tested if the human moDCs survived in the presence of the inhibitors and if we could detect decreased phagosomal acidification indicative of cross-presenting capacity. Human moDCs showed high survival (above 70%) in the presence of 0–40 µM of Wiskostatin and CK666 for up to 16 h (Fig. 2a). Phagosomal acidification of moDCs that had phagocytosed on bead were compared (Fig. 2b). CK666-treated moDCs, similar to mouse BM-DCs, showed decreased phagosomal acidification compared to non-treated moDCs (Fig. 2c). This data suggests that moDCs tolerate CK666 treatment and that CK666-treated moDCs have increased phagosomal acidification.

### CK666-treated BM-DCs express higher amounts of IL-2

**Fig. 3:**
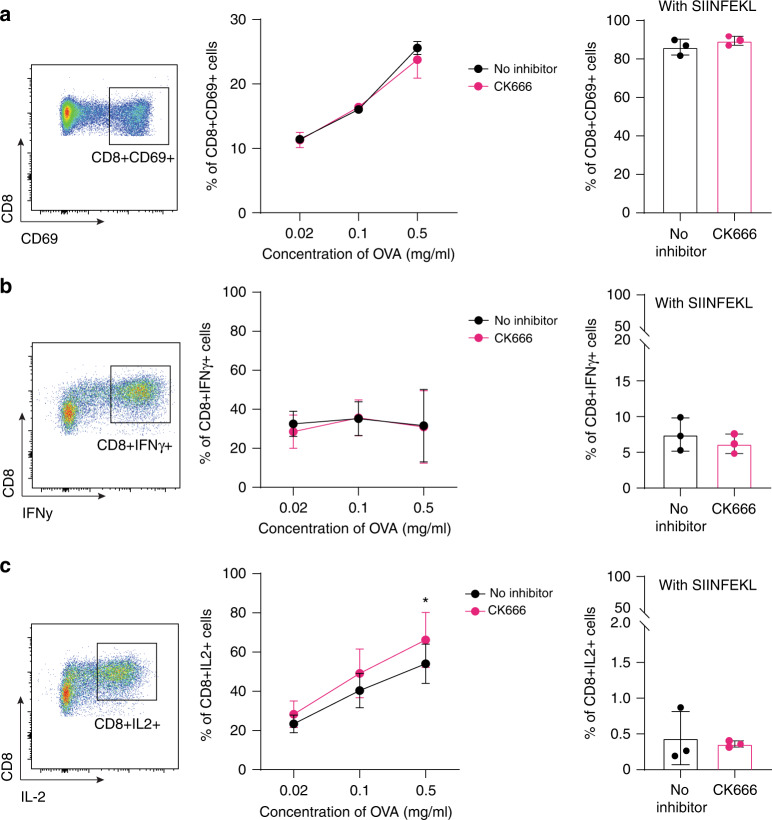
T-cell activation markers in vitro. **a** Representative plot and quantification of CD69 expression on CD8^+^ T cells when co-cultured with BM-DCs primed with different OVA concentrations from 0.02 to 0.5 mg/ml or 2 µg/ml of SIINFEKL and treated with CK666 (pink) or not (black). **b** Representative plot and quantification of IFNγ and **c** IL-2 production by CD8^+^ OT-1 T cells when co-cultured with BM-DCs primed with different OVA concentrations from 0.02 to 0.5 mg/ml or 2 µg/ml of SIINFEKL and treated with CK666 (pink) or not (black). Each dot corresponding to OVA concentration represents data pooled from 3 to 4 different experiments with 3 technical replicates each. Each dot in the SIINFEKL experiments represent 3 different biological replicates, each one with 3 technical replicates. Graphs show mean values ± SD and significance was assessed by two-way ANOVA with multiple comparisons. *p ≤ 0.05, **p ≤ 0.01, ***p ≤ 0.001, ****p ≤ 0.0001.

We next examined T-cell activation markers after DC:T-cell co-culture (gating strategy in Supplemental Fig. 1). The early activation marker CD69, and cytokines IFNγ and IL-2 production were measured using OVA at 0.02–0.5 mg/ml and SIINFEKL at 2 µg/ml. After 4 h of co-culture, CD8^+^ OT-1 T cells stimulated with CK666-treated and non-treated DCs had similar levels of CD69 (Fig. 3a). After 48 h of DC:T-cell co-culture time, IFNγ and IL-2 production by CD8^+^ OT-1 T cells was measured. IFNγ production at steady state and after PMA and Ionomycin stimulation was similar when comparing CD8^+^ OT-1 T cells stimulated with CK666-treated and non-treated DCs (Fig. 3b). However, CK666-treated BM-DCs induced higher IL-2 production by CD8^+^ OT-1 T cells when compared to non-treated BM-DCs (Fig. 3c), Non-treated and CK666-treated DCs loaded with SIINFEKL-OVA peptide alone induced only low cytokine production (Fig. 3c).

### CK666-treated BM-DCs induce more CD8^+^ T-cell proliferation in vivo

**Fig. 4:**
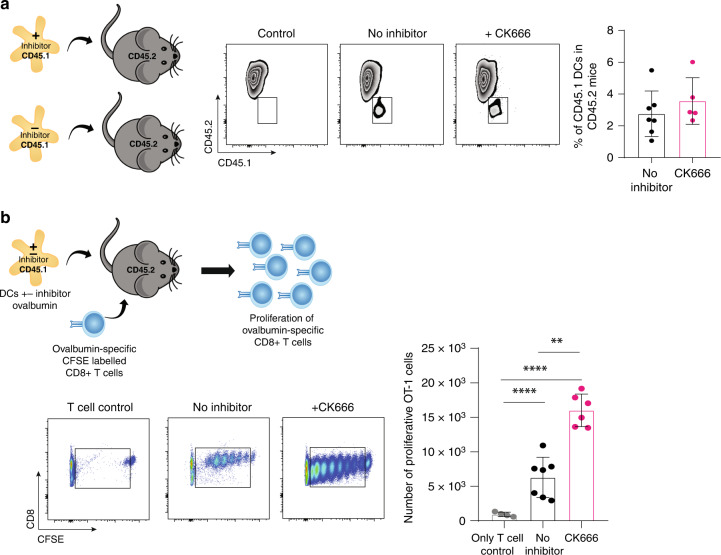
CK666-treated BM-DCs induce more CD8^+^ T-cell proliferation in vivo. **a** Experimental model of BM-DC migration; representative plots of CD45.1 BM-DCs detection on CD45.2 host mice and quantification of CD45.1 migratory CK666-treated (pink) or non-treated (black) BM-DCs. Results represent pooled data from 3 different experiments. Each dot represents one animal. **b** Experimental model of CD8^+^ OT-1 T-cell proliferation in vivo; representative plots of CFSE-labelled CD8^+^ OT-1 T-cell proliferation after primed by BM-DCs and quantification of CD8^+^ OT-1 T cells that proliferated after being activated by CK666-treated or non-treated BM-DCs in vivo. Results represent pooled data from 3 different experiments. Each dot represents one animal. Graphs show mean values ± SD and significance was assessed by two-way ANOVA with multiple comparisons. *p ≤ 0.05, **p ≤ 0.01, ***p ≤ 0.001, ****p ≤ 0.0001.

We next tested if DC treated with CK666 during uptake and processing of OVA ex vivo would induce the proliferation of CD8^+^ OT-1 T cells in vivo. We first examined if CK666 treatment of DCs would influence the migratory behaviour of DCs in vivo. BM-DCs from CD45.1 mice were injected into the footpad of CD45.2 mice. At 48 h, the draining popliteal LNs were collected to assess migratory CD45.1 BM-DCs by flow cytometry. CK666-treated and non-treated BM-DCs showed similar migration to the popliteal LNs (Fig. 4a). To test DC activation of CD8^+^ T-cell proliferation, we injected CK666-treated and non-treated BM-DCs primed with OVA and LPS into the footpad of CD45.2 mice. On the same day, we injected CFSE-labelled CD8^+^ OT-1 T cells intravenously. After 48 h, the popliteal LNs were collected and CFSE dilution and CD8^+^ OT-1 T-cell number was assessed by flow cytometry. As expected, the mice injected with only CD8^+^ OT-1 T cells, but no BM-DCs, did not show any T-cell proliferation. When compared to mice receiving non-treated BM-DCs, mice that received CK666-treated BM-DCs induced much higher CD8^+^ T-cell proliferation (Fig. 4b). This data show that CK666-treated BM-DCs are able to migrate to the draining lymph nodes, and they induce more CD8^+^ T-cell proliferation compared to non-treated BM-DCs.

### CK666-treated BM-DCs induce higher rejection of B16-mOVA melanoma cells

**Fig. 5:**
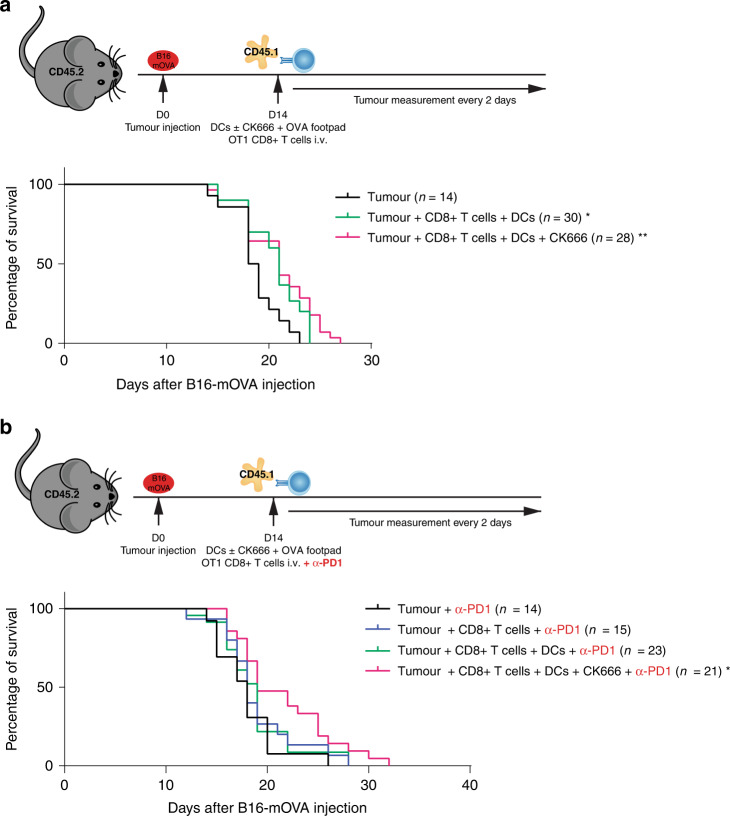
CK666-treated BM-DCs induce higher rejection of B16-mOVA melanoma tumours compared to non-treated DCs. **a** Experimental model of tumour rejection assay; survival curve of mice that only received OT-1 cell transfer (black line), mice that received non-treated BM-DCs and OT-1 cell transfer (green line) and mice that received CK666-treated BM-DCs and OT-1 cell transfer (pink line). Results represent pooled data from 6 different experiments and total number of mice used in each group is the following: control = 14, non-treated BM-DCs = 30, CK666-treated BM-DCs = 28. **b** Experimental model of tumour rejection assay with anti-PD1 150 µg/mouse; survival curve of mice that only received OT-1 cell transfer (black line), mice that received OT-1 cell transfer with anti-PD1 (blue line), mice that received non-treated BM-DCs, OT-1 cell transfer and anti-PD1 (green line) and mice that received CK666-treated BM-DCs, OT-1 cell transfer and anti-PD1 (pink line). Results represent pooled data from 4 different experiments and total number of mice used in each group is the following: control = 14, only T cells = 15, non-treated BM-DCs = 23, CK666-treated BM-DCs = 21. Graphs show mean values ± SD and significance was assessed by survival curve comparison using Mantel-Cox test. *p ≤ 0.05, **p ≤ 0.01, ***p ≤ 0.001, ****p ≤ 0.0001.

To test if CK666-treated BM-DCs had a higher capacity to activate and anti-tumour response, we performed in vivo tumour rejection experiments using a stable B16/F10 melanoma cell line expressing membrane-bound OVA (B16-mOVA) [43]. Mice were injected with B16-mOVA subcutaneously and tumours let grow for 14 days. At day 14, CK666-treated or non-treated BM-DCs primed with OVA and LPS were injected via the footpad and CD8^+^ OT-1 T cells injected intravenously. Tumour growth was monitored every 2 days until the tumour reached 1 cm^3^ when the mouse was sacrificed (Supplemental Fig. 2). Mice that only received T cells, and not BM-DCs, had the shortest survival. When comparing the groups that received CK666-treated BM-DCs or non-treated BM-DCs, mice receiving Ck666-treated BM-DCs survived 3 days longer (Fig. 5a). When combining checkpoint blockade anti-PD1with CD8^+^ OT-I T-cell injection, mice survival was extended in all groups with most prolonged survival of mice receiving CK666-treated DC (Fig. 5b).

## Discussion

In this study, we addressed the unmet need to promote cross-presentation by DCs in tumour therapy, thereby preventing the activation of T regulatory cells. DCs lacking WASp have increased cross-presentation and activation of cytotoxic CD8^+^ T cells [16]. Here we tested if small molecule inhibitors could mimic WASp deficiency to enhance DC cross-presentation. We established a protocol where BM-DCs were treated with the Arp2/3 inhibitor CK666 during 6 h of OVA uptake and processing, and thereafter the inhibitor was washed away before co-culture with CD8^+^ T cells or injection in vivo. This is important since CK666, other WASp signalling inhibitors such as Wiskostatin and ML141 or mutations in WASp interfere with cell proliferation and migration (this study and others [29–33, 44]). We found that this short incubation of BM-DCs with CK666 during ex vivo antigen uptake and processing reduced acidification and increased proliferation of antigen-specific CD8^+^ T cells in vitro and in vivo. To test the potential of the CK666 approach for DC-based cancer therapy, we used the aggressive B16 melanoma and found that mice receiving CK666-treated BM-DCs showed increased survival.

Small molecule inhibitors of the Arp2/3 complex have been well characterised and are considered highly specific for Arp2/3 as determined by crystal structure predictions. CK666 interacts with the pocket at the interface of Arp3 and Arp2, thereby inhibiting Arp2/3 complex activity [26, 42]. Moreover, DCs lacking Arpc2 or treated with shRNA for Arpc4 show a similar phenotype as CK666-treated DCs with increased migration in confined space caused by relocalization of polymerised actin away from the front in DCs [45]. Inhibitors of WASp signalling pathways, including CK666 has been widely tested in cancer therapies but only applied to kill cancer cells and to prevent migration and metastasis of cancer cells. Moreover, these studies have only been conducted in vitro, not considering toxicity in vivo [29–33]. Here we found that murine and human DCs are resistant to cell death upon treatment ex vivo with CK666 and Wiskostatin, at least for 16 h, which provides ample time for DC antigen uptake and processing. We therefore concluded that this approach is viable for DC therapy and tested this in vivo using the highly aggressive B16/F10-mOVA melanoma. We set up the model to mimic a real situation in which a patient with melanoma first detects a physical change, such as increased size, colour and shape of a mole. For this reason, we let the tumour grow for 14 days before applying the treatment. At this stage, melanoma starts to grow exponentially and is difficult to treat. We found that mice injected with CK666-treated BM-DCs had higher survival compared to mice injected with non-treated BM-DCs by 3 days. We think these results are highly significant and promising, considering that B16/F10-mOVA is an extremely aggressive tumour, and we are targeting it during the exponential phase of the tumour growth.

T-cell activation in vitro can be detected by the expression of receptors such as CD69 and the production of cytokines such as IL-2 and IFNγ. When comparing CK666-treated BM-DCs with non-treated BM-DCs, we show that CD69 and IFNγ levels were similar; however, IL-2 production was increased in CK666-treated BM-DCs. IL-2 is one of the most effective FDA-approved agents in the treatment of metastatic renal cell carcinoma and metastatic melanoma [46–48]. However, to maximise the therapy, nowadays, IL-2 is not administered alone but in combination with other anti-cancer immunotherapies [49]. One study involved 20 patients with malignant melanoma at stages III and IV that were treated with a DC-based vaccine combined with low doses of IL-2. In this study, four of seven patients from the group treated with combined DC therapy and IL-2 showed delayed type IV hypersensitive (DTH) reaction against melanoma cell lysates. Moreover, significant correlations were found between the DTH-positive responses and disease stability and patient survival [50]. Another study used DC-based vaccine combined with low doses of IL-2 is in phase I/II clinical trial for ovarian cancer patients. In this study, patients that had “no evidence of disease” status and received IL-2 treatment had increased overall survival. This suggests that DC-based vaccination induced tumour immunity that might be associated with long-term responses against ovarian cancer [51]. Together this data strengthen our hypothesis that modulation of DCs by CK666 could improve cancer immunotherapy. Combination treatments are the most common approach in cancer therapy. Cancer treatment includes surgery, sessions of chemotherapy, radiotherapy and nowadays, immunotherapy such as immune checkpoint blockades, including those targeting cytotoxic T lymphocyte-associated protein 4 (CTLA-4) and programmed cell death protein 1/programmed cell death ligand 1 (PD-1/PD-L1) [52–54]. For this reason, we think that our approach can be improved by combining CK666 with immune checkpoint blockade treatment. Moreover, a combination of small molecule inhibitors may be applicable, for example, by combining CK666 and Wiskostatin or CK666 combined with other CK drugs for Arp2/3 [55]. Together, our study demonstrates the potential of using the Arp2/3 inhibitor CK666 to enhance DC-based cancer therapy.

## Supplementary information

Suppl Figure 1-2 plus legends

## Data Availability

The authors confirm that all relevant data, materials and methods are included in the manuscript, figures and supplemental data.

## References

[1] Bevan MJ (1976). Cross-priming for a secondary cytotoxic response to minor h antigens with H-2 congenic cells which do not cross-react in the cytotoxic assay*. J Exp Med.

[2] Jung S, Unutmaz D, Wong P, Sano GI, De Los Santos K, Sparwasser T (2002). In vivo depletion of CD11c+ dendritic cells abrogates priming of CD8+ T cells by exogenous cell-associated antigens. Immunity..

[3] Steinman RM (1991). The dendritic cell system and its role in immunogenicity. Annu Rev Immunol.

[4] Kovacsovics-Bankowski M, Rock KL (1995). A phagosome-to-cytosol pathway for exogenous antigens presented on MHC class I molecules. Science.

[5] Rodriguez A, Regnault A, Kleijmeer M, Ricciardi-Castagnoli P, Amigorena S (1999). Selective transport of internalized antigens to the cytosol for MHC class I presentation in dendritic cells. Nat Cell Biol.

[6] Colbert JD, Cruz FM, Rock KL (2020). Cross-presentation of exogenous antigens on MHC I molecules. Curr Opin Immunol.

[7] Houde M, Bertholet S, Gagnon E, Brunet S, Goyette G, Laplante A (2003). Phagosomes are competent organelles for antigen cross-presentation. Nature..

[8] Guermonprez P, Saveanu L, Kleijmeer M, Davoust J, Van Endert P, Amigorena S (2003). ER-phagosome fusion defines an MHC class I cross-presentation compartment in dendritic cells. Nature..

[9] Shen L, Sigal LJ, Boes M, Rock KL (2004). Important role of cathepsin S in generating peptides for TAP-independent MHC class I crosspresentation in vivo. Immunity..

[10] Bertholet S, Goldszmid R, Morrot A, Debrabant A, Afrin F, Collazo-Custodio C (2006). Leishmania antigens are presented to CD8 + T cells by a transporter associated with antigen processing-independent pathway in vitro and in vivo. J Immunol.

[11] Li B, Hu L (2019). Cross-presentation of exogenous antigens. Transfus Clin Biol.

[12] Merzougui N, Kratzer R, Saveanu L, Van Endert P (2011). A proteasome-dependent, TAP-independent pathway for cross-presentation of phagocytosed antigen. EMBO Rep.

[13] Joffre OP, Segura E, Savina A, Amigorena S (2012). Cross-presentation by dendritic cells. Nat Rev Immunol.

[14] Fu C, Zhou L, Mi QS, Jiang A (2020). Dc-based vaccines for cancer immunotherapy. Vaccines..

[15] Wculek SK, Cueto FJ, Mujal AM, Melero I, Krummel MF, Sancho D (2020). Dendritic cells in cancer immunology and immunotherapy. Nat Rev Immunol.

[16] Baptista MAP, Keszei M, Oliveira M, Sunahara KKS, Andersson J, Kuo I (2016). Deletion of Wiskott–Aldrich syndrome protein triggers Rac2 activity and increased cross-presentation by dendritic cells. Nat Commun.

[17] Binks M, Jones GE, Brickell PM, Kinnon C, Katz DR, Thrasher AJ (1998). Intrinsic dendritic cell abnormalities in Wiskott-Aldrich syndrome. Eur J Immunol.

[18] Klos Dehring DA, Clarke F, Ricart BG, Huang Y, Gomez TS, Williamson EK (2011). Hematopoietic lineage cell-specific protein 1 functions in concert with the Wiskott–Aldrich syndrome protein to promote podosome array organization and chemotaxis in dendritic cells. J Immunol.

[19] De Noronha S, Hardy S, Sinclair J, Blundell MP, Strid J, Schulz O (2005). Impaired dendritic-cell homing in vivo in the absence of Wiskott-Aldrich syndrome protein. Blood..

[20] Machesky LM, Mullins RD, Higgs HN, Kaiser DA, Blanchoin L, May RC (1999). Scar, a WASp-related protein, activates nucleation of actin filaments by the Arp2/3 complex. Proc Natl Acad Sci USA.

[21] Thrasher AJ, Burns SO (2010). WASP: A key immunological multitasker. Nat Rev Immunol.

[22] Higgs HN, Pollard TD (1999). Regulation of actin polymerization by Arp2/3 complex and WASp/Scar proteins. J Biol Chem.

[23] Higgs HN, Pollard TD (2000). Activation by Cdc42 and PIP2 of Wiskott-Aldrich Syndrome protein (WASp) stimulates actin nucleation by Arp2/3 complex. J Cell Biol.

[24] Kim AS, Kakalis LT, Abdul-Manan N, Liu GA, Rosen MK (2000). Autoinhibition and activation mechanisms of the Wiskott-Aldrich syndrome protein. Nature..

[25] Oliveira MMS, Westerberg LS (2020). Cytoskeletal regulation of dendritic cells: an intricate balance between migration and presentation for tumor therapy. J Leukoc Biol.

[26] Nolen BJ, Tomasevic N, Russell A, Pierce DW, Jia Z, McCormick CD (2009). Characterization of two classes of small molecule inhibitors of Arp2/3 complex. Nature..

[27] Peterson JR, Bickford LC, Morgan D, Kim AS, Ouerfelli O, Kirschner MW (2004). Chemical inhibition of N-WASP by stabilization of a native autoinhibited conformation. Nat Struct Mol Biol.

[28] Surviladze Z, Waller A, Strouse JJ, Bologa C, Ursu O, Salas V, et al. A Potent and Selective Inhibitor of Cdc42 GTPase. In: Probe Reports from the NIH Molecular Libraries Program. 2010. 21433396

[29] Hong L, Kenney SR, Phillips GK, Simpson D, Schroeder CE, Nöth J (2013). Characterization of a Cdc42 protein inhibitor and its use as a molecular probe. J Biol Chem.

[30] Liu Z, Yang X, Chen C, Liu B, Ren B, Wang L (2013). Expression of the Arp2/3 complex in human gliomas and its role in the migration and invasion of glioma cells. Oncol Rep.

[31] Kazazian K, Go C, Wu H, Brashavitskaya O, Xu R, Dennis JW (2017). Plk4 promotes cancer invasion and metastasis through Arp2/3 complex regulation of the actin cytoskeleton. Cancer Res.

[32] Frugtniet BA, Martin TA, Zhang L, Jiang WG (2017). Neural Wiskott-Aldrich syndrome protein (nWASP) is implicated in human lung cancer invasion. BMC Cancer.

[33] Escudero-Esparza A, Jiang WG, Martin TA (2012). Claudin-5 is involved in breast cancer cell motility through the N-WASP and ROCK signalling pathways. J Exp Clin Cancer Res.

[34] Henson JH, Yeterian M, Weeks RM, Medrano AE, Brown BL, Geist HL (2015). Arp2/3 complex inhibition radically alters lamellipodial actin architecture, suspended cell shape, and the cell spreading process. Mol Biol Cell.

[35] Muul LM, Spiess PJ, Director EP, Rosenberg SA (1987). Identification of specific cytolytic immune responses against autologous tumor in humans bearing malignant melanoma. J Immunol.

[36] Dudley ME, Wunderlich JR, Robbins PF, Yang JC, Hwu P, Schwartzentruber DJ (2002). Cancer regression and autoimmunity in patients after clonal repopulation with antitumor lymphocytes. Science.

[37] Yee C, Thompson JA, Byrd D, Riddell SR, Roche P, Celis E (2002). Adoptive T cell therapy using antigen-specific CD8+ T cell clones for the treatment of patients with metastatic melanoma: In vivo persistence, migration, and antitumor effect of transferred T cells. Proc Natl Acad Sci USA.

[38] Hanson HL, Donermeyer DL, Ikeda H, White JM, Shankaran V, Old LJ (2000). Eradication of established tumors by CD8+ T cell adoptive immunotherapy. Immunity..

[39] Rosenberg SA, Yang JC, Restifo NP (2004). Cancer immunotherapy: moving beyond current vaccines. Nat Med.

[40] Zou W (2006). Regulatory T cells, tumour immunity and immunotherapy. Nat Rev Immunol.

[41] Melief CJM (2008). Cancer immunotherapy by dendritic cells. Immunity..

[42] Baggett AW, Cournia Z, Han MS, Patargias G, Glass AC, Liu SY (2012). Structural characterization and computer-aided optimization of a small-molecule inhibitor of the Arp2/3 complex, a key regulator of the actin cytoskeleton. ChemMedChem..

[43] DiLillo DJ, Yanaba K, Tedder TFB (2010). Cells are required for optimal CD4 + and CD8 + T cell tumor immunity: therapeutic B cell depletion enhances B16 melanoma growth in mice. J Immunol.

[44] Oliveira MMS, Kung SY, Moreau HD, Maurin M, Record J, Sanséau D (2022). The WASp L272P gain-of-function mutation alters dendritic cell coordination of actin dynamics for migration and adhesion. J Leukoc Biol.

[45] Vargas P, Maiuri P, Bretou M, Saéz PJ, Pierobon P, Maurin M (2016). Innate control of actin nucleation determines two distinct migration behaviours in dendritic cells. Nat Cell Biol.

[46] Rosenberg SA, Lotze MT, Muul LM, Leitman S, Chang AE, Ettinghausen SE (1985). Observations on the systemic administration of autologous lymphokine-activated killer cells and recombinant interleukin-2 to patients with metastatic cancer. N Engl J Med.

[47] Atkins MB, Lotze MT, Dutcher JP, Fisher RI, Weiss G, Margolin K (1999). High-dose recombinant interleukin 2 therapy for patients with metastatic melanoma: analysis of 270 patients treated between 1985 and 1993. J Clin Oncol..

[48] Dutcher J (2002). Current status of interleukin-2 therapy for metastatic renal cell carcinoma and metastatic melanoma. Oncology (Williston Park).

[49] Jiang T, Zhou C, Ren S (2016). Role of IL-2 in cancer immunotherapy. Oncoimmunology..

[50] Escobar A, López M, Serrano A, Ramirez M, Pérez C, Aguirre A (2005). Dendritic cell immunizations alone or combined with low doses of interleukin-2 induce specific immune responses in melanoma patients. Clin Exp Immunol.

[51] Baek S, Kim YM, Kim SB, Kim CS, Kwon SW, Kim YM (2015). Therapeutic DC vaccination with IL-2 as a consolidation therapy for ovarian cancer patients: a phase I/II trial. Cell Mol Immunol.

[52] Murciano-Goroff YR, Warner AB, Wolchok JD (2020). The future of cancer immunotherapy: microenvironment-targeting combinations. Cell Res.

[53] Leach DR, Krummel MF, Allison JP (1996). Enhancement of antitumor immunity by CTLA-4 blockade. Science.

[54] Iwai Y, Terawaki S, Honjo T (2005). PD-1 blockade inhibits hematogenous spread of poorly immunogenic tumor cells by enhanced recruitment of effector T cells. Int Immunol.

[55] Velle KB, Campellone KG (2018). Enteropathogenic E. coli relies on collaboration between the formin mDia1 and the Arp2/3 complex for actin pedestal biogenesis and maintenance. PLoS Pathog.

