# Supplementary material for: Increased cross-presentation by dendritic cells and enhanced anti-tumour therapy using the Arp2/3 inhibitor CK666
Source: Br J Cancer. 2023 Jan 11;128(6):982–91. doi: 10.1038/s41416-022-02135-4 (PMC10006228; doi:10.1038/s41416-022-02135-4)
Supplement: Supplementary file 1 — Suppl Figure 1-2 plus legends [file 41416_2022_2135_MOESM1_ESM.pdf]

Supplemental figure 1

A

Gatting strategy for CD8+ T cells proliferation in vitro

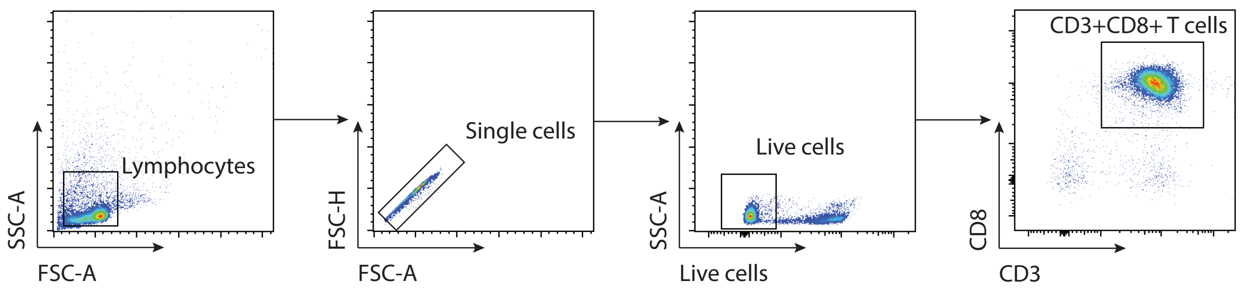

Supplemental figure 1. Gating strategy for CD8^{+} T cells proliferation in vitro.

Supplemental figure 2

A

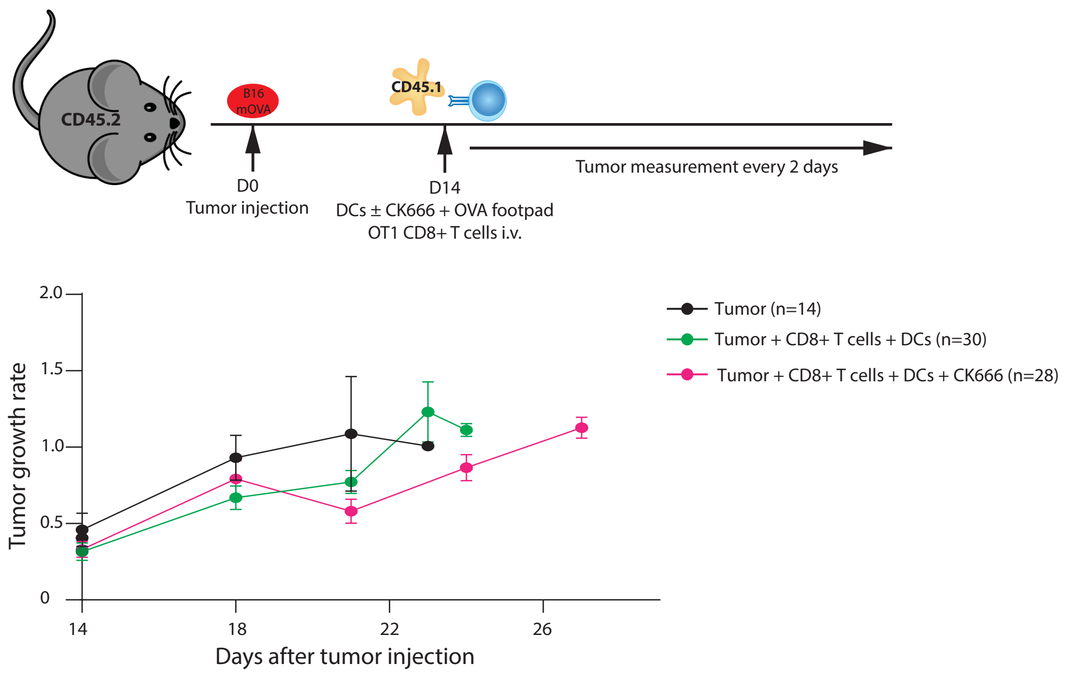

B

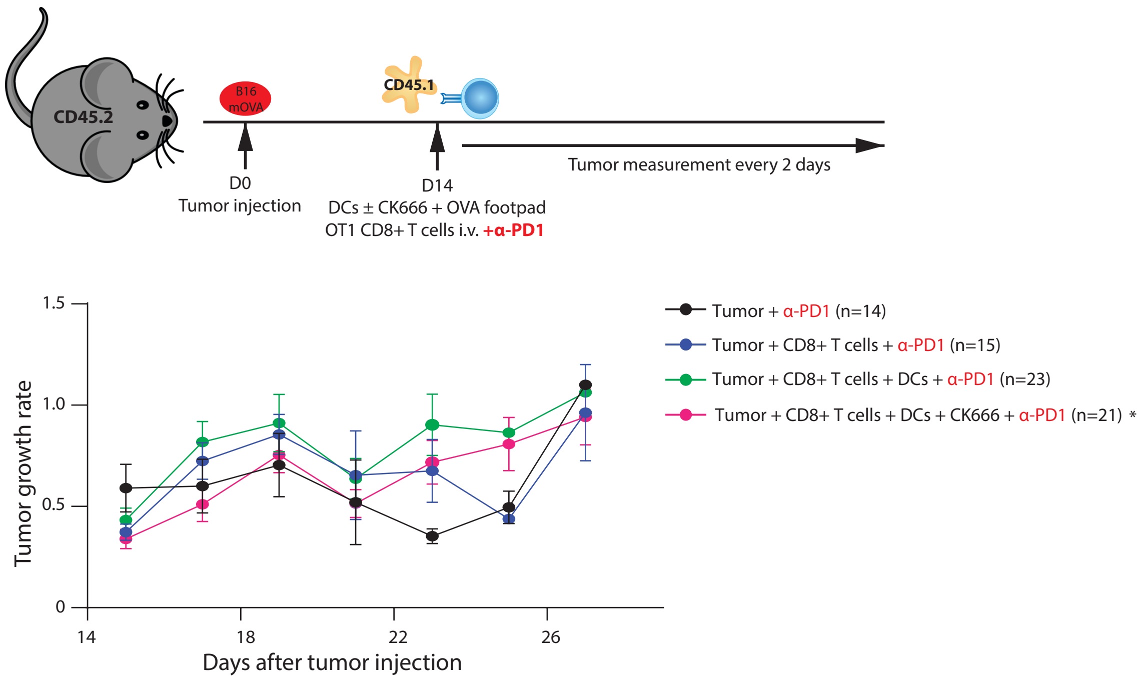

Supplemental figure 2: Tumor volume measurements. (**A**) Experimental model of tumor rejection assay; growth rate curves of tumor injected in mice that only received OT-1 cell transfer (black line), mice that received non-treated BM-DCs and OT-1 cell transfer (green line) and mice that received CK666-treated BM-DCs and OT-1 cell transfer (pink line). Results represent pooled data from 6 different experiments and total number of mice used in each group is the following: control=14, non-treated BM-DCs=30, CK666-treated BM-DCs=28. (**B**) Experimental model of tumor rejection assay with anti-PD1 150 µg/mouse; growth rate curves of tumor injected in mice that only received OT-1 cell transfer (black line), mice that received OT-1 cell transfer with anti-PD1 (blue line), mice that received non-treated BM-DCs, OT-1 cell transfer and anti-PD1 (green line) and mice that received CK666-treated BM-DCs, OT-1 cell transfer and anti-PD1 (pink line). Results represent pooled data from 4 different experiments and total number of mice used in each group is the following: control=14, only T cells=15, non-treated BM-DCs=23, CK666-treated BM-DCs=21.
